# Heterogeneity in the trypanosomosis incidence in Zebu cattle of different ages and sex on the plateau of eastern Zambia

**DOI:** 10.1016/j.actatropica.2007.05.011

**Published:** 2007-08

**Authors:** H. Simukoko, T. Marcotty, I. Phiri, J. Vercruysse, P. Van den Bossche

**Affiliations:** a University of Zambia, School of Veterinary Medicine, Zambia; b Institute of Tropical Medicine Antwerp, Animal Health Department, Nationalestraat 155, B-2000 Antwerp, Belgium; c Ghent University, Vakgroep Virologie, Parasitologie en Immunologie, Salisburylaan 133, B-9820 Merelbeke, Belgium; d Department of Veterinary Tropical Diseases, University of Pretoria, Onderstepoort, South Africa

**Keywords:** Trypanosomosis, Incidence, Endemic, Cattle, Heterogeneity, Zambia

## Abstract

On the plateau of eastern Zambia, trypanosomosis is endemic. *Glossina morsitans morsitans* Westwood (Diptera: Glossinidae), the only tsetse species present, is almost entirely dependent on livestock as its source of food with cattle being the most preferred host. To determine if tsetse challenge is distributed equally over the various age categories and sexes within a cattle herd, a longitudinal study of trypanosomosis incidence was conducted during the rainy season. A total of 354 head of cattle consisting of 40% oxen, 30% cows, 15% young stock, 13% calves and 2% bulls were sampled for three consecutive months and their infection statuses determined using the PCR–RFLP technique as diagnostic method. Results indicated that there were significant differences (*P* < 0.001) in the proportion of infected animals between the various categories. In oxen, the risk of infection was 5.6 times higher than in calves. Those results suggest heterogeneity in the challenge by tsetse flies and are in line with entomological observations on the feeding preference of tsetse on cattle. The implications of these results for the control of trypanosomosis in Eastern Province and other epidemiologically related areas are discussed.

## 1 Introduction

Livestock trypanosomosis is an important constraint to livestock productivity in sub-Saharan Africa (Swallow, 1998). It has adverse effects on rural development over vast areas (Holmes, 1997).

Over the years, a variety of methods to control the disease has been developed and used successfully for the area-wide or localised control of tsetse flies or the control of the parasite in susceptible animals. Currently, the control of animal trypanosomosis in eastern Zambia relies mainly on the curative or prophylactic treatment of livestock. The pattern of contact between haematophagous insects, such as tsetse flies, and their hosts is extremely heterogenous and non-random (Kelly, 2001). As a result, some host species are challenged substantially more than others and may contribute more to parasite transmission (Woolhouse et al., 1997). On the plateau of eastern Zambia, for example, a high proportion of tsetse feed on cattle rather than goats or pigs (Van den Bossche and Staak, 1997). Hence, the prevalence of trypanosome infections is likely to be substantially higher in cattle compared to the other livestock species. Moreover, much progress has been made in describing how some individuals within a species are bitten more than others (Torr et al., 2006; Torr and Mangwiro, 2000; Torr, 1994). In cattle, for example, tsetse are attracted most to animals that produce large amounts of host odours. Hence, a host's attractiveness to tsetse flies is correlated with its weight and/or age (Torr et al., 2006). Although odours may be important in attracting tsetse flies to individual hosts, they may be a less important determinant of biting risk when animals of all sizes, ages and sexes are aggregated in a herd or in a kraal. This is usually the case under traditional livestock management practices. This study was conducted to determine if under such traditional cattle management practices challenge differs between animals of different age and sex and kept in the same herd.

## 2 Materials and methods

### 2.1 Study area

The study was carried out in an area situated between 31°47′ and 31°55′ E, and between 13°55′ and 14°12′ S in Katete District, Eastern Province, Zambia. It is a highly cultivated area with a cattle population of approximately 8–10 animals/km^2^ (based on an aerial survey conducted in August 1997). *Glossina morsitans morsitans*, which takes the majority (75%) of its bloodmeals on cattle, is the only tsetse species present (Van den Bossche and Staak, 1997; Van den Bossche and De Deken, 2002). Bovine trypanosomosis (mainly due to *Trypanosoma congolense*) is endemic with an average herd prevalence of about 30% (personal observation).

The annual climatic cycle comprises three seasons; the warm rainy season (from early November to late April), the cold dry season (from early May to late August) and the hot dry season (from early September to late October).

### 2.2 Animal selection and follow-up

Cattle sampled during the longitudinal study were part of 19 herds of which all owners were based in Alick village (31°52′E and 14°06′S). The herds were selected because they grazed in the same grazing areas surrounding the village. A total of 354 animals was identified for the study. This sample size provided 95% certainty of detecting at least one positive case at a prevalence of 5% (Cannon and Roe, 1982). To select animals from the 19 herds to be included in the study, a proportional stratified random sampling was applied in which age and sex categories were considered as strata. Random sampling was performed in such a way that the number of samples in each stratum was proportional to the normal herd structure in the study area (Doran, 2000). A total of 354 animals were identified. They consisted of approximately 40% oxen, 30% adult females (cows, >48 months), 15% young females and young males (12 to 48 months), 13% calves (<12 months) and 2% bulls (>48 months) and were kept under traditional livestock management practices. During the study period all categories of cattle (including calves) were herded together. At the start of the experiment, all sampled animals were ear-tagged and injected intramuscularly with a double dose (7.0 mg/kg bw) of diminazene aceturate (Berenil^®^, Hoechst) to clear all trypanosomal infections. Through offering free diagnosis and treatment farmers were likely to abide by the request not to treat their cattle. Blood was collected from all sampled animals on a monthly basis and the trypanosome infection status of each animal was determined. Blood collection started in March (one and a half months after the diminazene treatment) and continued for three consecutive months (March–May), a period when tsetse challenge in the study area is high (Van den Bossche and De Deken, 2002). Animals infected with trypanosomes were treated with a curative dose of diminazene aceturate (at 3.5 mg/kg bw).

### 2.3 Sampling and diagnosis

From each animal, jugular blood was collected in a vacutainer tube with EDTA as anticoagulant. After sampling, the vacutainer tubes were placed in a coolbox containing ice packs. From each vacutainer tube, blood was collected into two capillary tubes which were sealed at one end with “Cristaseal” (Hawksley, Lancing, UK). The capillary tubes were span in a microhaematocrit centrifuge for 5 min at 9000 rpm. The buffy coats of the two capillary tubes were extruded onto a labelled filter paper (Whatman no. 3, Whatman^®^). Filter papers were stored in sealed plastic bags containing silica gel at −18 °C. The samples were further analysed using the PCR–RFLP described by Geysen et al. (2003).

### 2.4 Statistical analysis

Statistical analyses were carried out in Stata Corp. (2003). For the survival analysis, a Cox proportional hazards model was used to compare the relative risks of infection in the different age and sex categories. Infected animals were censored. The proportional-hazards assumption was tested on Schoenfeld residuals (*P* > 0.05).

## 3 Results

**Fig. 1:**
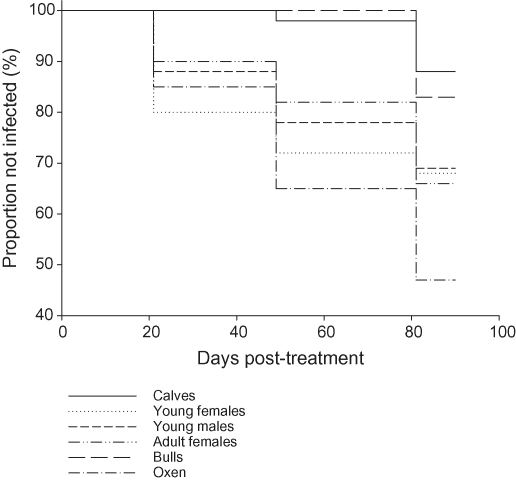
Kaplan-Meier survival curves for infection with *T. congolense* of cattle of different ages and sex on the plateau of eastern Zambia.

During the 3 months observation period, a total of 180 sentinel animals became infected with trypanosomes (all *T. congolense*). The monthly incidence of new infections during each month of observation in each category and the relative risks to infection for each category are shown in Table 1. The survival analysis showed significant differences in the risk of infection between age categories and sexes (Fig. 1). The risk of infection relative to calves was almost six times higher in oxen (*P* < 0.001) and almost twice as high in cows (*P* = 0.01) and young animals (*P* = 0.05). The risk of infection in bulls was not significantly different from that in calves (*P* < 0.8). Finally, the risk of infection was lower in cows than in oxen (*P* = 0.01).

## 4 Discussion

The results of this longitudinal study show significant heterogeneity of trypanosomosis incidence in the various cattle categories. Although the study is of relative short duration, the timing was chosen because of the high level of trypanosomosis challenge during the rainy season and the communal herding during this time of the year. Moreover, during the observation period (peak of the rainy season) oxen are less used for animal traction and are, most of the time, kept within the herd. The study's results are in accordance with the entomological findings on the attractiveness of different age categories and sexes of cattle to tsetse flies. Indeed, according to Torr et al. (2006) and Torr and Mangwiro (2000), tsetse flies are attracted significantly more by odour of large animals (i.e. oxen) and animals that showed less defensive behaviour and least by calves. The latter is especially so when calves are part of a herd. This study suggests that the observed differences in attractiveness also translate in differences in trypanosomosis incidence even when animals are kept in a herd.

A number of studies have shown an effect of age on the incidence or prevalence of trypanosome infections in cattle. Rowlands et al. (2001) found a significant effect of age on the incidence of *T. congolense* infections in animals below 15 months of age with calves being the least infected. Similar low infection rates in calves were observed by Trail et al. (1994). In both cases, however, calves were kept at the homestead. Furthermore, although those studies confirm part of our observations, the findings suffer from the poor sensitivity of the pararasitological diagnostic tests used to identify trypanosome infections.

The low prevalence and incidence of trypanosome infections in calves has also been attributed to the protective effect of maternal immunity that does not prevent calves from becoming infected but mitigates the adverse effect of such an infection by reducing the parasitaemia to a very low almost undetectable level (Fiennes, 1970). Although maternal immunity cannot be excluded, the low incidence of trypanosome infections in calves suggests low levels of challenge or low attractiveness to tsetse. The low risk of infection in bulls is attributed to the small sample size.

Heterogeneity of incidence or individual variation in exposure to vector-borne diseases is a well-known phenomenon and may have repercussions for the optimal design of disease control programs (Kelly, 2001; Woolhouse et al., 1997). Although the results obtained from this study may not be sufficient to draw general conclusions for the control of livestock trypanosomosis, some important observations can be made with regard to the control of bovine trypanosomosis in the study area and perhaps in other areas where epidemiological circumstances are comparable. First, the high risk of trypanosome infection in oxen and the high proportion of oxen in the cattle herd make oxen the most appropriate part of the herd for prophylactic treatment with trypanocidal drugs. Indeed, such targeted drug campaigns will affect the most infected part of the herd, protects the most valuable part of the herd in the mixed-crop livestock production system and may, at the same time, reduce the number of tsetse that will become infected. In addition, diminishing the trypanocidal drug use frequency is likely to reduce the risk of resistance development in trypanosomes. Second, because of the high attractiveness of oxen even when part of a herd, oxen may be the most appropriate cattle category to be treated with insecticides to control tsetse.

## Figures and Tables

**Table 1:** Monthly incidence of new trypanosome infections and relative risks (CI) of infections, with calves being used as reference category, in cattle of different ages and sex on the plateau of eastern Zambia

Category	Number of animals	Incidence (in %)			Relative risks (CI)
		Month			
		1	2	3	
Calves	46	0	6.5	8.7	
Young females	28	25	18	7.1	3.3 (1.1–10.1)
Young males	28	14.3	21.4	10.7	3.1 (1–9.8)
Cows	106	11.3	14.2	17	3.3 (1.3–8.5)
Bulls	6	0	0	16.7	1.4 (0.16–11.8)
Oxen	140	19.3	28.6	23.6	5.6 (2.3–14.1)
